# S100A9 Regulates MDSCs-Mediated Immune Suppression via the RAGE and TLR4 Signaling Pathways in Colorectal Carcinoma

**DOI:** 10.3389/fimmu.2019.02243

**Published:** 2019-09-18

**Authors:** Mao Huang, Rui Wu, Lu Chen, Qi Peng, Shue Li, Yan Zhang, Lan Zhou, Liang Duan

**Affiliations:** ^1^Key Laboratory of Laboratory Medical Diagnostics, Ministry of Education, Department of Laboratory Medicine, Chongqing Medical University, Chongqing, China; ^2^Department of Laboratory Medicine, The First Affiliated Hospital of Chongqing Medical University, Chongqing, China; ^3^Department of Academic Research, The Second Affiliated Hospital of Chongqing Medical University, Chongqing, China; ^4^Department of Laboratory Medicine, The Second Affiliated Hospital of Chongqing Medical University, Chongqing, China

**Keywords:** CRC, S100A9, MDSCs, migration, activation

## Abstract

Myeloid-derived suppressor cells (MDSCs) are a major component of the immunosuppressive tumor microenvironment (TME) and have been recognized as a contributing factor to inflammation-related cancers. However, the molecular mechanisms of MDSCs accumulation and activation remain elusive. We previously showed that the proinflammatory molecule S100A9 in TME exerts a tumor-promoting effect in colorectal carcinoma (CRC). In this report, we investigated the effect and molecular mechanisms of S100A9 on the accumulation and immunosuppressive function of MDSCs in CRC. Elevated S100A9 and MDSCs were found in tumor tissue and peripheral blood from CRC patients. Circulating S100A9 and MDSCs were positively associated to each other, and both S100A9 and MDSCs were correlated to neoplastic progression. Using a CRC cell line LoVo-induced MDSCs model, we found that S100A9 stimulated chemotaxis and activation but not viability of MDSCs. Mechanistic studies demonstrated that activation of RAGE-mediated p38 MAPK and TLR4-mediated NF-κB signaling pathways were involved in S100A9-induced chemotaxis and MDSCs activation, respectively. Furthermore, ROC analysis showed that combination detection of S100A9 and MDSCs was superior to individual detection of these two factors for diagnosing CRC patients with advanced staging and lymphatic metastasis, which yielded an area under the ROC curve (AUC) of 0.92 with 86.7% sensitivity and 86.4% specificity, and an AUC of 0.82 with 75% sensitivity and 77.1% specificity, respectively. Collectively, our study suggests that the S100A9 plays a pivotal role in immunosuppressive TME by stimulating MDSCs chemotaxis and activation, and combination detection of S100A9 and MDSCs may serve as a potential marker for diagnosis of CRC progression.

## Introduction

Colorectal carcinoma (CRC) is the third most common cancer and the fourth leading cause of cancer death worldwide (1, 2). Inflammatory bowel disease (IBD) is associated with an increased risk of CRC. A growing body of studies has suggested that inflammation plays an important role in initiation of colitis-associated CRC (3). However, the immune status in the inflammatory microenvironment and the underlying mechanisms related to immune escape in CRC remain unknown.

Myeloid-derived suppressor cells (MDSCs) represent a heterogeneous population of immature myeloid cells consisting of precursors for granulocytes, macrophage, and dendritic cells that are major components of the immunosuppressive TME (4). Accumulation of MDSCs has been thought to be a significant factor linking inflammation and cancer (5). Human MDSCs are characterized by the myeloid marker HLA-DR^−^CD33^+^CD11b^+^ (6). This tolerogenic appearance of MDSCs represents a common trait of cancer and other non-cancerous diseases such as sepsis, bacterial, viral, and parasitical infections, autoimmune diseases, and aging (5–7). As one type of the most potent immunosuppressive cells, MDSCs facilitate tumor progression by suppressing T cell response, blocking natural killer cell activation, limiting dendritic cell maturation, and inducing regulatory T (Treg) cell generation (5), which are associated with high expression and secretion of immunosuppressive molecules such as interleukin (IL)-10, arginase-1(Arg-1), inducible nitric oxide synthase (iNOS) and ROS (5–7). The accumulation of MDSCs in TME involves multifarious mechanisms including trafficking, expansion and activation in different types of cancer (3, 8–13). Increased circulating and tumor-infiltrating MDSCs are also found in CRC, which correlates to cancer progression (14, 15). However, the exact molecular mechanism of MDSCs accumulation and activation in CRC remains elusive.

S100A9 belongs to a family of intracellular EF-hand motif calcium-binding proteins found exclusively in vertebrates. S100A9 is constitutively expressed in myeloid cells including granulocytes, monocytes, early-differentiation cells of the myeloid lineage, and cancer cells (16). It has been shown that S100A9 overexpression is correlated to invasion and metastasis in various cancers, and S100A9 directly enhances tumor cell malignancy by activating TLR4-mediated or RAGE-mediated signaling cascades (17–19). Our previous study, consistent with others, showed that S100A9 in the CRC microenvironment directly contributes to malignancy in CRC cancer cells (20, 21). Recently, S100A9 was shown to modulate inflammatory or immune cell migration and activation through TLR4-mediated or RAGE-mediated signaling pathways (22–25). Given that MDSCs are precursors for inflammatory and immune cells, we hypothesized that S100A9 regulates trafficking, expansion and activation of MDSCs to establish an immunosuppressive microenvironment to potentiate CRC progression.

In this study, we investigated the association between MDSCs and S100A9 in CRC tumor tissues and peripheral blood, and the effect of molecular mechanisms of S100A9 on trafficking, cell vitality, and activation of MDSCs in CRC. We found that both MDSCs and S100A9 are correlated to Dukes staging and lymph node metastasis, and activation of the RAGE-mediated p38 MAPK and TLR4-mediated NF-κB signaling pathways are involved in S100A9-induced trafficking and activation of MDSCs, respectively. Combination detection of S100A9 and MDSCs may be a serum marker for CRC diagnosis, particularly for CRC staging and metastasis. Our results highlight the significance of S100A9 in regulating MDSCs in the immunosuppressive microenvironment and implicates that S100A9 could be a potential therapeutic target for CRC.

## Materials and Methods

### Patient and Sample Collection

Whole blood samples from CRC patients (*n* = 52) and healthy controls (*n* = 30) were collected from the Second Affiliated Hospital of Chongqing Medical University from September 2015 to August 2017. The clinicopathological data of the subjects including gender, age, tumor location, Dukes staging, cell differentiation, and metastasis at initial diagnosis are shown in Table 1. Serum samples from 4 ml of coagulated blood by centrifugation were immediately separated and frozen at −80°C until use. Peripheral blood mononuclear cells (PBMCs) were isolated from whole blood via Ficoll-Hypaque gradient centrifugation. Clinical histological proven CRC tissue samples (*n* = 16) and the matched distal normal tissues (n = 16) were collected from patients who underwent surgical resection at the Second Affiliated Hospital of Chongqing Medicine University. All of the patients and healthy donors provided written informed consent before sampling.

**Table 1:** The characteristics of enrolled individuals.

**Parameters**	**CRC (*n* = 52)** ***n*, %**	**HC (*n* = 30)** ***n*, %**
**Gender**		
Male (*n*, %)	34 (65.38%)	15 (50%)
Female (*n*, %)	18 (34.62%)	15 (50%)
**Age**		
<60 (*n*, %)	30 (57.69%)	20 (66.6%)
≥60 (*n*, %)	22 (42.31%)	10 (33.3%)
**Location**		
Colon (*n*, %)	25 (48.08%)	NA
Rectum (*n*, %)	27 (51.92%)	NA
**Tumor differentiation**		
Low (*n*, %)	30 (57.69%)	NA
High/middle (*n*, %)	22 (42.31%)	NA
**Dukes staging**		
A/B (*n*, %)	24 (46.15%)	NA
C/D (*n*, %)	28 (53.85%)	NA
**Lymphatic metastasis**		
Absent (*n*, %)	36 (69.23%)	NA
Present (*n*, %)	16 (30.77%)	NA

*NA, not available*.

### Antibodies, Inhibitors, and Preparation of the Recombinant Proteins

The antibodies included anti-S100A9 (Cat no. ab92507; Abcam), anti-TLR4 (Cat no. sc-293072; Santa Cruz Biotechnology), anti-RAGE (Cat no. sc-80653; Santa Cruz Biotechnology), anti-p38 (Cat no. 9212; Cell Signaling Technology), anti-p65 (Cat no. 3034; Cell Signaling Technology), anti-ERK1/2 (Cat no. 4695; Cell Signaling Technology), anti-JNK (Cat no. 9253; Cell Signaling Technology), anti-AKT (Cat no. 8596; Cell Signaling Technology), anti-phospho(p)-p38 (Cat no. 4511; Cell Signaling Technology), anti-p-p65 (Cat no. 3033; Cell Signaling Technology), anti-p-ERK1/2 (Cat no. 3510; Cell Signaling Technology), anti-p-JNK (Cat no. 4668; Cell Signaling Technology), anti-p-AKT (Cat no. 9271; Cell Signaling Technology), anti-CD8 (Cat no. 340046, BD), anti-HLA-DR (Cat no. 4310370, eBioscience), anti-CD33 (Cat no. 4296343, eBioscience) and CD11b (Cat no. 4291932, eBioscience), and horseradish peroxidase-conjugated anti-mouse, anti-rabbit IgG antibodies. The inhibitors contained TAK-242 (MedChemExpress, New Jersey), FPS-ZM1 (MedChemExpress, New Jersey), SB203580 (Beyotime) and BAY 11-7082 (Beyotime). The preparation of the recombination GST-S100A9 protein, as well as its control protein GST, have previously been described (21).

### Cells Culture

The human CRC LoVo cells were cultured in Dulbecco's modified Eagle's medium (DMEM; Gibco, Life Technologies) supplemented with 10% fetal bovine serum (FBS; HyClone), 100 U/ml of penicillin and 100 μg/ml of streptomycin. The LoVo- induced MDSCs were cultured in 10% fetal bovine serum, 100 U/ml of penicillin and 100 μg/ml of streptomycin 1640 medium (Gibco, Life Technologies). The cell culture was maintained at 37°C in a humid atmosphere containing 5% CO_2_.

### ELISA Assay

Serum levels of S100A9, Arg-1, and iNOS were measured using a commercially available enzyme-linked immunosorbent assay by ELISA kit (ELISA LAB, Wuhan, China) according to the manufacturer's instructions.

### Single Cell Suspension

The collected CRC and paraneoplastic tissues were washed with PBS three times and then cut into pieces, and enzymatically digested with type I collagenase (Sigma, USA) for 1~2 h at 37°C with mixing every 20 min. The resulted single cell samples were used for flow cytometry (FCM) analysis.

### Immunohistochemistry (IHC)

Immunohistochemical staining for CD33 and S100A9 was performed using anti-human CD33 (Cat no. ab92507, eBioscience) and an anti-S100A9 (Cat no. ab92507, Abcam) antibodies following the manufacturer's instructions. Briefy, the deparaffinized and dehydrated sections were boiled for 10 min in 0.01 M citrate buffer and incubated with 0.3% hydrogen peroxide (H_2_O_2_) in methanol for 15 min to block endogenous peroxidase, incubated with primary and peroxidase-tagged secondary antibodies sequentially, and colorized with 0.05% 3,3-diaminobenzidine tetrachloride (DAB). The sections were counterstained with hematoxylin, and observed, and representative images were captured under an inverted phase contrast microscope (Olympus B640, Japan).

### Western Blot

The cells were collected and washed with ice-cold PBS and lysed on ice in radio immunoprecipitation assay (RIPA) buffer. An equal amount of proteins of the samples was separated in 10% SDS-PAGE and blotted onto PVDF membranes. The membranes were blocked with 5% bovine serum albumin (BSA) and incubated with the primary antibodies and horseradish peroxidase-conjugated secondary antibodies. The proteins of interest were detected using the SuperSignal West Pico Chemiluminescent Substrate kit. The results were recorded using the Bio-Rad Electrophoresis Documentation (Gel Doc 1000) and Image Lab version software.

### Flow Cytometry (FCM) Analysis

Human monoclonal Abs against HLA-DR-PE (Cat no. 4310370, eBioscience), CD33-FITC (Cat no. 4296343, eBioscience), and CD11b-APC (Cat no. 4291932, eBioscience) conjugated with different fluorescent dyes were used for FCM analysis. Immunophenotyping of circulating or tumor-infiltrating MDSCs were classified as HLA-DR^−^ CD33^+^ CD11b^+^ cells via FCM staining using the multiplex gating strategy.

Human monoclonal Abs against Arg1-Alexa Fluor 488 (Cat no. 53369782, invitrogen) and iNOS (Cat no. MA517139, Invitrogen) were used for FCM analysis for Arg1 and iNOS expression in MDSCs. Samples were analyzed on a BECKMAN COULTER Navios FCM, and the data were analyzed using the Flowjo software.

### Induction of Tumor-Associated MDSCs *in vitro*

CD33^+^ cells were separated from mixed PBMCs from different CRC patients using human CD33 MicroBeads (Cat no. 18257, STEMCELL Technologies Inc) according to the manufacturer's instructions. Isolated CD33^+^ cells were co-cultured with LoVo cells in 6-well plates in a Transwell System (0.4 μm pore, Corning) at a ratio of 1:3 for 48 h. CD33^+^ cells cultured in medium alone were included as a control. The LoVo-induced MDSCs markers in the resulted cells were analyzed by FCM.

### CD8^+^ T Cells Proliferation Suppression Assay

For the analysis of suppressing CD8^+^ T cells proliferation by MDSCs, PBMCs from healthy donors were labeled with carboxyfluorescein diacetate succinimidyl ester (CFSE, 10 μM), seeded in OKT3-coated 96-well plates and co-cultured with LoVo-induced MDSCs at a 1:2 ratio for 3 days, stained with an anti-human CD8 mAb followed by FCM analysis. Individually cultured CFSE-labeled PBMCs were used as a control.

### Chemotaxis Assay

Chemotaxis assay was performed using 24-well plates with 5-μm-pore size inserts (Corning) according to the manufacturer's instructions. A total of 1 × 10^6^ MDSCs in serum-free medium were loaded into the upper chamber and GST and GST-S100A9 proteins were in the lower chamber in the presence or absence of specific inhibitors. After incubation for 24 h, migrated cells were counted in the upper chamber.

### RNA Isolation and Real-Time PCR Analysis

The LoVo-induced MDSCs were stimulated with GST and GST-S100A9 proteins for 24 h and total RNA was extracted from cells using Trizol (Invitrogen) in accordance with the manufacturer's instructions. Reverse transcription-PCR was done using the PrimeScript^TM^ RT Reagent Kit (Takara, Japan) and TB Green^TM^ Premix Ex Taq^TM^ II (Takara, Japan). The sequences of primers were: GAPDH primers: (forward) 5′-CAGCGACACCCACTCCTC-3′ and (reverse) 5′-TGAGGTCCACCACCCTGT-3′; Arg-1 primers: (forward) 5′-GTTTCTCAAGCAGACCAGCC-3′ and (reverse) 5′-GCTCAAGTGCAGCAAAGAGA-3′; iNOS pimers: (forward) 5′-CAGCGGGATGACTTTCCAA-3′ and (reverse) 5′-AGGCAAGATTTGGACCTGCA-3′; IL-10 primers: (forward) 5′-GGCTTCCTAACTGCTACA-3′ and (reverse) 5′-CTCCTGACCTCAAGTGAT-3′; TLR4 primers: (forward) 5′-AGAATGCTAAGGTTGCCGCT-3′ and (reverse) 5′-CTATCACCGTCTGACCGAGC-3′; RAGE primers: (forward) 5′-ACTACCGAGTCCGTGTCTACC-3′ and (reverse) 5′-GGAACACCAGCCGTGAGTT-3′. Reactions were performed in triplicate using SYBR Green master mix (Takara, Japan) and normalized to GAPDH using the ΔΔCt method.

### ROS Detection

ROS was measured with the Reactive oxygen detection kit (Beyotime, Jiangsu, China). LoVo-induced MDSCs (1 × 10^5^) were seeded in 96-well plates and the probe (DCFH-DA) was loaded into the cells according to the manufacturer's instructions. Then the DCFH-DA loaded MDSCs were cultured in the cell incubator for 30 min and washed 3 times with serum-free medium to eliminate the residual probe. The recombination proteins and specific inhibitors were added to the labeled MDSCs for 30 min, then the fluorescence was measured by a fluorescence microplate reader.

### Cell Viability Analysis

To detect the effect of S100A9 on viability of LoVo-induced MDSCs, the cells (1 × 10^5^ cells/well) were grown in triplicates in 96-well plates and treated with or without GST or GST-S100A9 for 12, 24, and 36 h and cell viability was analyzed by CCK8 assay according to the manufacture's instruction. CCK8 reagent was added into the medium at the indicated time. After a 1 h incubation under the culture condition, absorbance at 450 nm was measured on a microplate reader.

### Statistical Analysis

All the numerical data were presented as means ± standard deviation. All the statistical analyses were performed using GraphPad Prism 6 (GraphPad Software). One-way ANOVA followed by the S-N-K test, was used for the analyses of quantitative RT-PCR, cell viability, and transwell migration. The Mann-Whitney test was used for clinical data, such as the expression of S100A9, Arg1, and iNOS in serum. The Spearman test was used to analyze the relationship of S100A9 with MDSCs, Arg-1, and iNOS. The ROC analysis was used to prove the diagnostic power for S100A9, MDSCs, and their combination in CRC progression. Significant probability values were indicated as ^*^p < 0.05, ^**^p < 0.01, ^***^p < 0.001.

## Results

### Increased Number of Tumor-Infiltrating and Circulating MDSCs in CRC Patients

**Figure 1:**
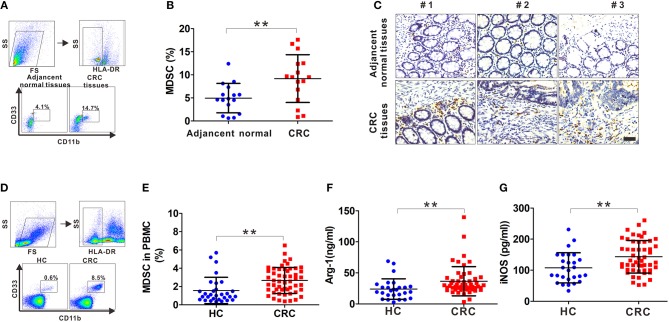
The frequency of MDSCs in CRC patients. **(A)** The gating strategy for HLA-DR^−^CD33^+^CD11b^+^ cells from tissue samples of CRC patients. **(B)** Statistical analysis for the percentage of infiltrating MDSCs in 16 paired CRC samples including tumor tissues and adjacent normal tissues. **(C)** Representative IHC staining for CD33 in tumor tissues and adjacent normal tissues from three CRC patients. Blank scale bars = 100 μm. **(D)** The gating strategy for HLA-DR^−^CD33^+^CD11b^+^ cells from the peripheral blood of CRC patients. **(E)** Statistical analysis for the percentage of circulatory MDSCs in PBMCs from healthy controls (HC, *n* = 30) and CRC patients (*n* = 52) by FCM. **(F–G)** ELISA analysis of serum Arg1 and iNOS levels from HC (*n* = 30) and CRC patients (*n* = 52). Data represents the mean±SD. **p < 0.01.

Fresh tumor tissues and adjacent normal tissues collected from CRC patients were used to generate single cell samples, and MDSCs were detected by FCM using the myeloid marker HLA-DR^−^CD33^+^CD11b^+^. There was a significant increase in MDSCs in CRC compared with that in the adjacent tissues (Figures 1A,B). Also, the infiltrating CD33^+^ cells were prominently increased in the CRC tissues (Figure 1C). Next, we determined the number of MDSCs in peripheral blood. As expected, there was increased MDSCs circulating in the CRC compared with the healthy controls (Figures 1D,E). Furthermore, the serum levels of Arg-1 and iNOS, the immunosuppressive molecules mainly expressed and secreted by MDSCs for suppressing T cell function, were measured. Arg-1 and iNOS were at high levels in the serum of CRC patients compared to that in healthy controls (Figures 1F,G), indicating that the accumulated MDSCs were in an activated state. Altogether, these results suggest that both infiltrating MDSCs in tumor tissues and circulating MDSCs are increased in CRC patients.

### Elevated S100A9 Expression in CRC

**Figure 2:**
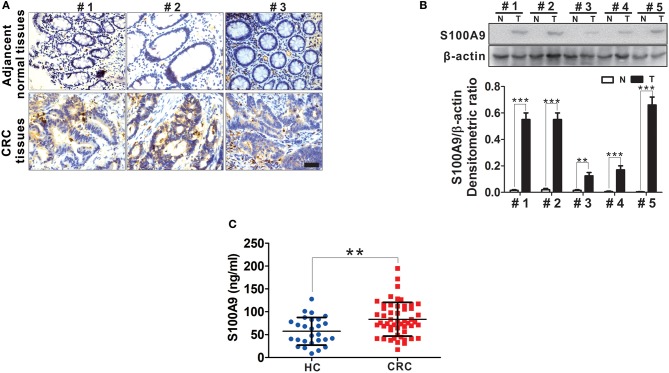
The expression of S100A9 protein in CRC. **(A)** Representative IHC staining for S100A9 expression in tumor tissues and adjacent normal tissues from three CRC patients. Blank scale bars = 100 μm. **(B)** Western blot analysis of S100A9 expression in five randomly selected CRC patients' tumor tissues and adjacent normal tissues. N, adjacent normal tissues; T, tumor tissues. β-actin served as a loading control. **(C)** ELISA analysis of serum S100A9 level from HC (*n* = 30) and CRC (*n* = 52) patients. Data represents the mean±SD. **p < 0.01, ***p < 0.001.

S100A9 expression in tumor and adjacent normal tissues from CRC patients was detected by IHC. We observed increased S100A9 expression, which was mainly localized to the cytoplasm, in CRC tissues compared with adjacent normal tissues (Figure 2A). Additionally, increased S100A9 expression in CRC was detected in five randomly selected CRC patients' tumor tissues and adjacent normal tissues by Western blot (Figure 2B). Similarly, serum S100A9 levels in CRC patients were markedly higher than that in healthy controls (Figure 2C). All these data suggest that S100A9 expression is elevated in CRC tissues and sera.

### Increased S100A9 Expression and MDSCs Number Are Associated With Neoplastic Progression of CRC

**Figure 3:**
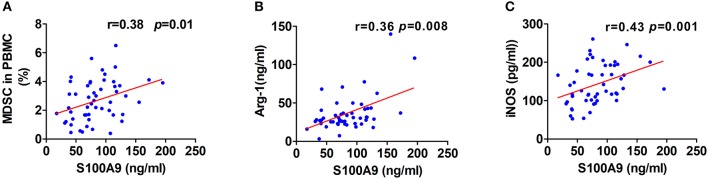
Correlation of serum S100A9 levels with MDSCs frequency or immunosuppressive molecules Arg-1 and iNOS. **(A–C)** Correlation of serum S100A9 levels and MDSCs frequency in PBMC **(A)**, serum Arg-1 **(B)**, and iNOS **(C)** in CRC patients (*n* = 52). *p < 0.05, **p < 0.01, ***p < 0.001.

Although one previous report showed high levels of serum S100A9 in CRC (26), the association of S100A9 levels in the neoplastic properties of CRC has not been studied. We found that S100A9 expression was correlated to Dukes staging and metastasis status but not to tumor location and histological differentiation. Moreover, there was a similar result in the relationship between MDSC numbers and Dukes staging and metastasis status but not tumor location and histological differentiation (Table 2). These findings imply that the increased S100A9 and MDSCs are closely related to Dukes staging and the metastasis of CRC. Additionally, serum S100A9 levels and MDSC numbers were positively correlated to each other in CRC patients (Figure 3A), and there was also a positive correlation of serum S100A9 levels with Arg-1 and iNOS levels, two immunosuppressive molecules mainly expressed and secreted by MDSCs, in CRC (Figures 3B,C).

**Table 2:** Relationship between MDSCs frequency or serum S100A9 levels and clinicopathological parameters of CRC patients.

**Variables**	**MDSCs**		**S100A9**	
	**Mean ± SD (%)**	***p*-value**	**Mean ± SD (ng/ml)**	***p*-value**
**Location**				
Colon	2.851 ± 1.607	0.498	87.58 ± 37.034	0.621
Rectum	2.488 ± 1.245		79.981 ± 37.589	
**Differentiation**				
Low	2.806 ± 1.283	0.744	82.826 ± 33.713	0.807
High	2.557 ± 1.625		84.139 ± 43.010	
**Dukes staging**				
A/B	1.887 ± 1.127	0.000	66.734 ± 30.764	0.002
C/D	3.327 ± 1.332		96.028 ± 36.970	
**Lymphatic metastasis**				
Negative	2.294 ± 1.340	0.005	73.967 ± 33.184	0.005
Positive	3.491 ± 1.296		103.537 ± 37.880	

### S100A9 Effectively Stimulates CRC-Associated MDSC Chemotaxis and Activation *in vitro*

**Figure 4:**
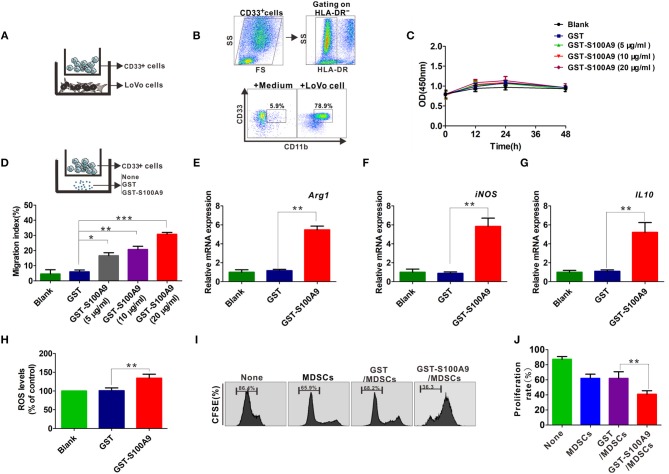
The influence of S100A9 in LoVo-induced MDSCs vitality, chemotaxis and activation *in vitro*. **(A)** The CD33^+^ cells were separated from PBMCs with CD33 positive magnetic beads, and co-cultured with LoVo cells to induce into CRC-associated MDSCs *in vitro*. **(B)** Identification of LoVo induced-MDSCs characterized by the myeloid marker with HLA-DR^−^CD33^+^CD11b^+^ by FCM. CD33^+^ cells cultured in medium alone were included as a control. **(C)** CCK8 assay for cell viability of LoVo-induced MDSCs treated with different concentrations of GST-S100A9 (5, 10, and 20 μg/ml) and GST proteins for 12, 24, and 48 h. **(D)** Chemotaxis assay for LoVo-induced MDSCs treated with different concentrations of GST-S100A9 (5, 10, and 20 μg/ml) and GST proteins for 24 h. **(E–G)** Real-time PCR analysis for mRNA expression of immunosuppressive molecules, including *Arg*-*1* **(E)**, *iNOS* **(F)**, and *IL*-*10* **(G)** in LoVo-induced MDSCs after GST-S100A9 (20 μg/ml) and GST (20 μg/ml) protein treatment for 24 h. **(H)** Fluorescence intensity analysis for ROS production in LoVo-induced MDSCs treated with GST-S100A9 (20 μg/ml) and GST (20 μg/ml) proteins. **(I)** Suppression of LoVo-induced MDSCs treated with GST-S100A9 (20 μg/ml) and GST (20 μg/ml) proteins on T cells *in vitro*. **(J)** A statistical graph of the suppressive effect of LoVo-induced MDSCs treated with GST-S100A9 (20 μg/ml) and GST (20 μg/ml) proteins on CD8^+^ T cells. Data represent three independent experiments and are represented as Mean±SD. *p < 0.05, **p < 0.01, ***p < 0.001.

CD33^+^ cells from peripheral blood could be differentiated into MDSCs by co-culture with cancer cells including CRC cell lines HCT116 and SW480 *in vitro* (8, 14). Here, CD33^+^ cells were co-cultured with another CRC cell line, LoVo (Figure 4A), which had a stronger metastasis potential for 48 h and the cell phenotypes were further examined by FCM, showing that the proportion of HLA-DR^−^CD33^+^CD11b^+^ MDSCs was markedly higher when CD33^+^ cells were co-cultured with LoVo compared with CD33^+^ cells cultured in medium alone (Figure 4B). We then investigated the possible regulatory effects of S100A9 on cell vitality, chemotaxis and activation of MDSCs. There was no detectable effect of recombinant GST-S100A9 protein at different concentrations on the vitality of LoVo-induced MDSCs (Figure 4C). In contrast, GST-S100A9 significantly elevated MDSCs migration index in a concentration-dependent manner, suggesting that S100A9 promotes MDSCs chemotaxis (Figure 4D). GST-S100A9 (20 μg/ml) also remarkably up-regulated mRNA levels of the immunosuppressive molecules *Arg-1, iNOS*, and *IL-10* increased ROS production (Figures 4E–H). S100A9-induced increased protein levels of the Arg-1 and iNOS were also confirmed (Figure S1). In addition, PBMCs isolated from the peripheral blood of healthy donors were labeled with CFSE and co-cultured with S100A9-stimulated MDSCs, and T cell proliferation was examined. The results showed that S100A9 (20 μg/ml) potentiated the suppressing effect of MDSCs on T cell proliferation (Figures 4I,J). Altogether, these results imply that S100A9 can stimulate traffic and activate MDSCs but not cell vitality, and enhances the suppressing effect of MDSCs on T cell proliferation.

### RAGE and TLR4 Are Involved in S100A9-Mediated MDSCs Chemotaxis and Activation

**Figure 5:**
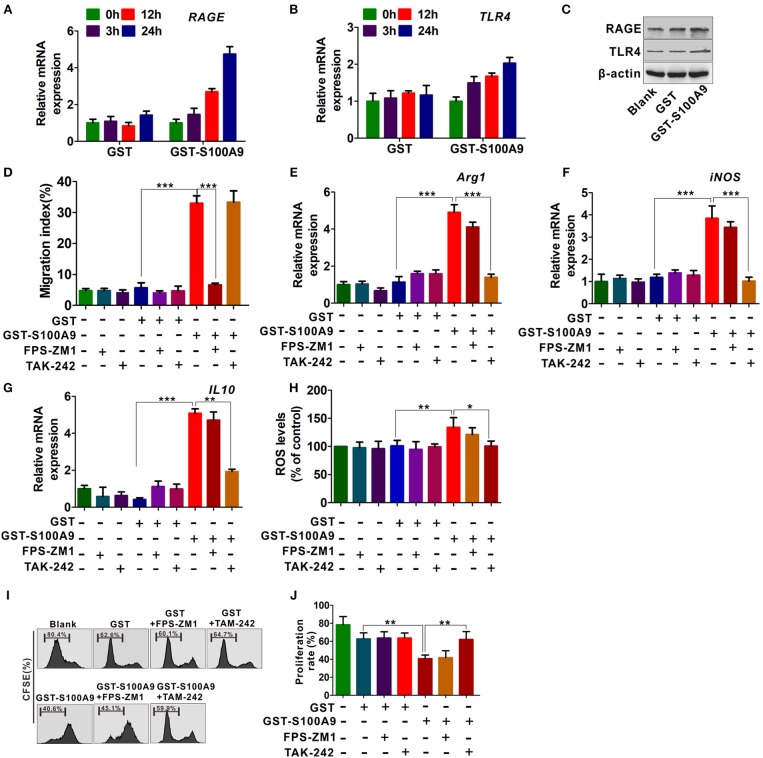
RAGE and TLR4 receptors are responsible for S100A9-mediated MDSCs chemotaxis and activation. **(A,B)** Real-time quantitation PCR analysis of *RAGE* **(A)** and *TLR4* **(B)** mRNA expression in LoVo-induced MDSCs treated with GST-S100A9 (20 μg/ml) and GST (20 μg/ml) for 3, 12, and 24 h. **(C)** Western blot analysis of RAGE and TLR4 expression in MDSCs treated with GST-S100A9 (20 μg/ml) and GST (20 μg/ml) for 24 h. **(D)** Chemotaxis assay for LoVo-induced MDSCs pretreated with and without inhibitors FPS-ZM1 (100 nM) and TAK-242 (100 nM) for 30 min followed by stimulation with GST-S100A9 (20 μg/ml) and GST (20 μg/ml) proteins for 24 h. **(E–G)** Real-time quantitative PCR analysis for mRNA expression of immunosuppressive molecules, including *Arg1* **(E)**, *iNOS* **(F)**, and *IL10* **(G)** in LoVo-induced MDSCs pretreated with and without inhibitors FPS-ZM1 (100 nM) and TAK-242 (100 nM) for 30 min followed by stimulation with GST-S100A9 (20 μg/ml) and GST (20 μg/ml) proteins for 24 h. **(H)** Fluorescence intensity analysis for ROS production in LoVo-induced MDSCs pretreated with and without inhibitors FPS-ZM1 (100 nM) and TAK-242 (100 nM) for 30 min followed by stimulation with GST-S100A9 (20 μg/ml) and GST (20 μg/ml) proteins for 24 h. **(I)** Suppression of LoVo-induced MDSCs pretreated with and without inhibitors FPS-ZM1 (100 nM) and TAK-242 (100 nM) for 30 min followed by stimulation with GST-S100A9 (20 μg/ml) and GST (20 μg/ml) proteins on T cells *in vitro*. **(J)** A statistical graph of the suppressive effect of LoVo-induced MDSCs pretreated with and without inhibitors FPS-ZM1 (100 nM) and TAK-242 (100 nM) for 30 min followed by stimulation with GST-S100A9 (20 μg/ml) and GST (20 μg/ml) proteins on CD8^+^ T cells. Data represent three independent experiments and are represented as Mean±SD. *p < 0.05, **p < 0.01, ***p < 0.001.

To further investigate the mechanisms by which S100A9 enhances MDSCs migration and activation, we focused on the RAGE and TLR4 that are the most common S100A9 receptors (16, 17, 27). GST-S100A9 (20 μg/ml) up-regulated mRNA levels of *TLR4* and *RAGE* in MDSCs in a time-dependent manner (Figures 5A,B). Consistently, the protein levels of TLR4 and RAGE in MDSCs were enhanced by GST-S100A9 (20 μg/ml) (Figure 5C). The RAGE inhibitor FPS-ZM1, but not the TLR4 TAK-242, inhibited the migration index of the S100A9-treated MDSCs (Figure 5D). In contrast, TAK-242, but not FPS-ZM1, blocked S100A9-induced mRNA expression of *Arg1, iNOS*, and *IL-10* and ROS production in MDSCs (Figures 5E–H). Consistent with these results, the suppressive effect of S100A9-treated MDSCs on CD8^+^ T cell proliferation was also inhibited by TAK-242 but not FPS-ZM1 (Figures 5I,J). These results suggest that RAGE and TLR4 are responsible for S100A9-mediated MDSCs chemotaxis and activation, receptively.

### Activation of p38 and NF-κB Signaling Are Responsible for S100A9-Induced MDSCs Chemotaxis and Activation

**Figure 6:**
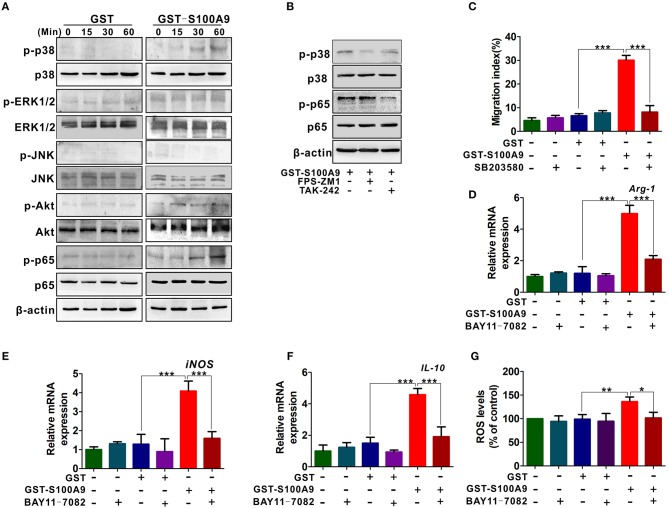
RAGE-mediated p38/MAPK and TLR4-mediated NF-κB are responsible for the chemotaxis and activation of MDSCs resulted by S100A9. **(A)** Western blot analysis of p-p38, p-ERK, p-JNK, p-AKT, and p-p65 expression in LoVo-induced MDSCs treated by GST-S100A9 (20 μg/ml) or GST (20 μg/ml) for 0, 15, 30, and 60 min. **(B)** Western blot analysis of p-p38 and p-p65 expression in GST-S100A9-treated MDSCs with inhibitors FPS-ZM1 (100 nM) and TAK-242 (100 nM) for 60 min. **(C)** Chemotaxis assay for LoVo-induced MDSCs pretreated with and without inhibitor SB203580 (50 nM) for 30 min followed by stimulation with GST-S100A9 (20 μg/ml) and GST (20 μg/ml) proteins for 24 h. **(D–F)** Real-time PCR analysis for mRNA expression of immunosuppressive molecules *Arg-1* **(D)**, *iNOS* **(E)**, and *IL-10* **(F)** in LoVo-induced MDSCs pretreated with and without inhibitor BAY11-7082 (50 nM) for 30 min followed by stimulation with GST-S100A9 (20 μg/ml) and GST (20 μg/ml) proteins for 24 h. **(G)** Fluorescence intensity analysis for ROS production in LoVo-induced MDSCs pretreated with and without inhibitor BAY11-7082 (50 nM) for 30 min followed by stimulation with GST-S100A9 (20 μg/ml) and GST (20 μg/ml) proteins for 24 h. Data represent three independent experiments and are represented as Mean±SD. *p < 0.05, **p < 0.01, ***p < 0.001.

RAGE and TLR4 mediate multiple signaling pathways such as mitogen-activated protein kinase (MAPK), PI3K/Akt, and nuclear factor-kappa B (NF-κB) pathways for inflammation and cancer (28–30). We then focused on these signaling pathways and explored their possible roles in S100A9-mediated MDSCs chemotaxis and activity. P-p38 and p-p65 levels but not p-ERK1/2, p-JNK and p-Akt were increased in MDSCs (Figure 6A). FPS-ZM1 and TAK-242 blocked S100A9-induced p-p38 and p-p65, respectively (Figure 6B), suggesting that RAGE and TLR4 are involved in S100A9-mediated p38 MAPK and NF-κB pathways, respectively. Exposure of GST-S100A9-treated MDSCs to the p38 inhibitor SB203580 decreased the migration index of GST-S100A9-treated MDSCs (Figure 6C). On the contrary, exposure of GST-S100A9-treated MDSCs to the NF-κB inhibitor BAY11-7082 decreased the mRNA levels of *Arg1, iNOS*, and *IL-10* and ROS (Figures 6D–G). Altogether, these results indicate that RAGE-mediated p38 MAPK and TLR4-mediated NF-κB are responsible for the chemotaxis and activation of MDSCs induced by S100A9, respectively.

### Diagnostic Power of Serum S100A9 and MDSCs of Peripheral Blood for CRC Neoplastic Progression

**Figure 7:**
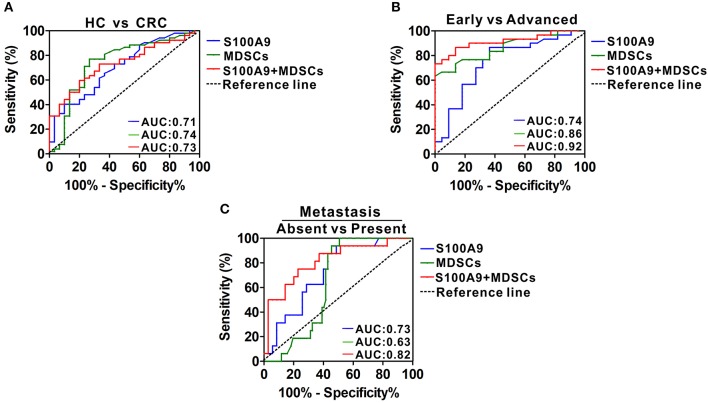
Diagnostic power of serum S100A9 and MDSCs frequency for CRC progression. **(A)** ROC curves of serum S100A9, MDSCs, and their combination for detecting CRC. **(B)** ROC curves of serum S100A9, MDSCs and their combination for identifying advanced stages from early stages in CRC patients. **(C)** ROC curves of serum S100A9, MDSCs, and their combination for detecting metastasis from none in CRC patients.

We investigated the potential value of serum S100A9 and MDSCs of peripheral blood for CRC progression. ROC analysis showed that the diagnostic value of S100A9, MDSCs and their combination detection yielded an AUC of 0.71, 0.74, and 0.73 (Figure 7A, Table 3), suggesting that S100A9 and MDSCs, individually or in combination, are weak in discriminating CRC patients from healthy individuals. We next evaluated whether they can distinguish early and advanced stages of CRC. A combination of S100A9 and MDSCs had a better detection efficiency than S100A9 or MDSCs alone, which yielded an AUC of 0.92 with 86.7% sensitivity and 86.4% specificity (Figure 7B, Table 3). Furthermore, we found that the combination was superior to S100A9 or MDSCs alone in identifying CRC patients with lymphatic metastasis, which yielded an AUC of 0.82 with 75.0% sensitivity and 77.1% specificity (Figure 7C, Table 3). Those results imply that combination detection of S100A9 and MDSCs could be a serum marker for CRC diagnosis in disease stage and metastasis.

**Table 3:** The efficacy analysis of the detection index.

	**HC vs. CRC**			**Early vs. Advanced**			**Absent vs. Present**		
	**S100A9**	**MDSCs**	**Combination**	**S100A9**	**MDSCs**	**Combination**	**S100A9**	**MDSCs**	**Combination**
AUC	0.71	0.74	0.73	0.75	0.86	0.92	0.72	0.74	0.82
Sensitivity	73.1%	73.30%	66.7%	86.70%	73.30%	86.7%	93.8%	68.8%	75%
Specificity	56.1%	76.9%	73.10%	63.60%	81.8%	86.4%	51.40%	80%	77.1%

## Discussion

**Figure 8:**
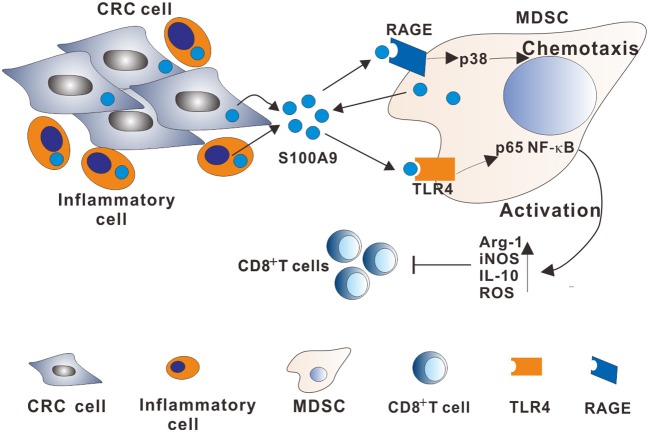
A model describing the mechanisms of the S100A9-induced immunosuppressive microenvironment by regulating MDSCs chemotaxis and activation in CRC. Increased extracellular S100A9 protein derived from CRC cells, inflammatory cells, or MDSCs in TME stimulates RAGE-dependent p38 MAPK signaling cascade, promoting MDSCs chemotaxis. Additionally, increased extracellular S100A9 in TME stimulates the TLR4-dependent NF-κB signaling cascade, promoting MDSCs activation by upregulating immunosuppressive molecules Arg-1, iNOS, and IL10 expression, and ROS production.

MDSCs are immune-modulatory cells that suppress adaptive immunity to promote tumor progression and metastasis. Although the immunosuppressive role of MDSCs in the tumor niche is documented, the detailed regulatory mechanisms by which MDSCs are recruited and activated have not been well-elucidated. S100A9 have gained interest because it functions as a chemokine for regulating inflammatory cell or immunocyte, which creates a proinflammatory microenvironment to facilitate tumor growth and metastasis. Here, we provide data suggesting a regulatory role of S100A9 in the CRC microenvironment on MDSCs chemotaxis and activation involving RAGE-dependent p38 MAPK and TLR4-dependent NF-κB signaling pathways (Figure 8).

In humans, MDSCs constitute a heterogeneous cell population that is not well-characterized, partially because no unified markers are currently available for these cells. Most studies concur with the observation that MDSCs express CD11b and CD33 but lack the expression of mature myeloid cell markers such as HLA-DR (5). In this study, we identified and characterized the MDSC population in CRC patients and found a strong correlation between MDSCs and CRC neoplastic progression, which is consistent with previous findings in other cancers such as bladder cancer (8), melanoma (31), and hepatocellular carcinoma (32). Our data also demonstrated that high levels of immunosuppression molecules Arg-1 and iNOS mainly expressed in MDSCs in CRC serum samples, demonstrating that MDSCs may not only be significantly increased in CRC but also have more suppressive function compared to those from healthy donor cells with the same phenotype.

We further demonstrated that MDSCs from CRC patients or induced by LoVo cells exerted the MDSCs phenotype, which was characterized by expressing CD11b^+^ CD33^+^ HLA-DR^−^. The results that show that the CRC cells can induce functional MDSCs *in vitro* agrees with previous studies for other types of cancer cells (14, 33, 34). Various tumor-derived factors have been reported to induce immature myeloid cells (CD33^+^ cells) to differentiate to MDSCs, these factors include prostaglandin E2, IL-6, IL-10, IL-1β, TGF-β, and proangiogenic factors such as vascular endothelial growth factor (35, 36). The proinflammatory S1008 and S100A9 proteins were reported to regulate MDSC accumulation in all regions of dysplasia and adenoma in a colitis-associated colon cancer model (37). Therefore, we speculate that the induction of MDSCs from CD33^+^ PBMCs may be associated with these factors derived by LoVo cells, which needs future studies.

Elevated S100A9 expression in CRC tissues and its association with disease progression have been reported in our previous study (21). Here, similar results were obtained. In addition, we explored the relationship of serum S100A9 levels with CRC neoplastic progression. Interestingly, recent research indicated that S100A9 could be one marker for circulatory MDSCs (38), and other studies showed that MDSCs from tumor-bearing mice or peripheral blood in cancer patients express and secrete S100A9 in an autocrine manner (37). Here, a positive correlation between S100A9 and MDSCs as well as immunosuppressive molecules Arg1 or iNOS was also observed. Consistent with the literature, our present findings further suggest that S100A9 participates in immunosuppression during CRC development by regulating MDSCs.

MDSCs have emerged as key effector cells in the immunosuppressive microenvironment of many solid tumor malignancies, and several factors that infuence MDSC recruitment and function have been investigated. For example, tumor-derived granulocyte-macrophage colony-stimulating factor recruits and alters MDSC proliferation and function in pancreatic ductal adenocarcinoma, thwarting CD8^+^ T cell-mediated anti-tumor immunity (39). Accumulation of CCL2 was found to be correlated to poor prognosis in glioblastoma patients, whereas deficiency of CCL2 reduced the recruitment of MDSCs and Treg cells in a glioblastoma mouse model (40). CXC-motif chemokines such as CXCL12 and IL-8 are also involved in the recruitment of MDSCs (41, 42). Considering the regulatory effect of S100A9 on chemotaxis and activation for inflammatory cells shown in previous studies (43, 44), we assessed whether the protein is responsible for the chemotaxis or activation of MDSCs. Our data showed that S100A9 could intensify the recruitment and function of MDSCs, suggesting that S100A9 plays an important role in the immunosuppressive microenvironment by regulating MDSCs. RAGE-mediated or TLR4-mediated downstream signal cascades have been reported to be involved in the migration or activation function of inflammatory cells induced by S100A9 in inflammation (22, 45–47). Here, we found that the RAGE-mediated p38 and TLR4-mediated NF-κB signaling pathways were involved in MDSC chemotaxis and activation, respectively.

Over the past few decades, increasing experimental evidence has demonstrated that either S100A9 or MDSCs contribute to tumor development (5, 16), suggesting that the detection of S100A9 and/or MDSCs in peripheral blood may be a diagnostic and progression prediction marker for CRC. While the differentiating power of S100A9, MDSCs or their combination is weak for CRC diagnosis, the combination detection could a marker for predicting CRC stages and lymph node metastasis. All this evidence suggests that the combination of S100A9 and MDSCs could be a candidate marker to detect CRC neoplastic progression.

In conclusion, the current observations indicate that accumulative MDSCs and increased S100A9 in CRC patients contribute to immune suppression. A positive correlation of MDSCs and S100A9 was observed in the peripheral blood of CRC patients. Both MDSCs and S100A9 were correlated to CRC neoplastic progression, which could be a candidate marker to detect CRC neoplastic progression. We further demonstrated that S100A9 plays a role in MDSCs recruitment and activation in CRC by regulating the RAGE-mediated p38 MAPK and TLR4-mediated NF-κB signaling pathways. Thus, inhibiting MDSCs by targeting S100A9-induced signaling pathways may be a beneficial option for CRC patients.

## Data Availability Statement

The datasets generated for this study are available on request to the corresponding author.

## Ethics Statement

The serum and tissue samples were obtained from CRC patients with no chemotherapy or radiotherapy prior to surgery. Written informed consent, was obtained from all of these participants. This study was approved by the Research Ethics Committee of the Second Affiliated Hospital of Chongqing Medical University.

## Author Contributions

MH and RW performed the experiments, analyzed the data, and wrote the manuscript. LC, QP, YZ, and SL analyzed the data. LD and LZ conceived the study ideas, oversaw the research, and co-wrote the manuscript.

### Conflict of Interest

The authors declare that the research was conducted in the absence of any commercial or financial relationships that could be construed as a potential conflict of interest.

## References

[1] Choi Y Sateia HF Peairs KS Stewart RW Screening for colorectal cancer. Semin Oncol. (2017) 44:34–44. 10.1053/j.seminoncol.2017.02.002 28395761

[2] Wang K Karin M. Tumor-elicited inflammation and colorectal cancer. Adv Cancer Res. (2015) 128:173–96. 10.1016/bs.acr.2015.04.014 26216633

[3] Chen J Pitmon E Wang K. Microbiome, inflammation and colorectal cancer. Semin Immunol. (2017) 32:43–53. 10.1016/j.smim.2017.09.006 28982615

[4] Groth C Hu X Weber R Fleming V Altevogt P Utikal J. Immunosuppression mediated by myeloid-derived suppressor cells (MDSCs) during tumour progression. Br J Cancer. (2019) 120:16–25. 10.1038/s41416-018-0333-1 30413826 PMC6325125

[5] Umansky V Blattner C Gebhardt C Utikal J. The role of Myeloid-Derived Suppressor Cells (MDSC) in cancer progression. Vaccines. (2016) 4:36. 10.3390/vaccines4040036 27827871 PMC5192356

[6] Filipazzi P Huber V Rivoltini L. Phenotype, function and clinical implications of myeloid-derived suppressor cells in cancer patients. Cancer Immunol Immunother. (2012) 61:255–63. 10.1007/s00262-011-1161-9 22120756 PMC11029611

[7] Zhang H Lian M Zhang J Bian Z Tang R Miao Q. A functional characteristic of cysteine-rich protein 61: modulation of myeloid-derived suppressor cells in liver inflammation. Hepatology. (2018) 67:232–46. 10.1002/hep.29418 28777871

[8] Zhang H Ye YL Li MX Ye SB Huang WR Cai TT. CXCL2/MIF-CXCR2 signaling promotes the recruitment of myeloid-derived suppressor cells and is correlated with prognosis in bladder cancer. Oncogene. (2017) 36:2095–104. 10.1038/onc.2016.367 27721403

[9] Ma S Cheng Q Cai Y Gong H Wu Y Yu X IL-17A produced by γδT cells promotes tumor growth in hepatocellular carcinoma. Cancer Res. (2014). 74:1969–82. 10.1158/0008-5472.CAN-13-2534 24525743

[10] Ouyang L Chang W Fang B Qin J Qu X Cheng F. Estrogen-induced SDF-1α production promotes the progression of ER-negative breast cancer via the accumulation of MDSCs in the tumor microenvironment. Sci Rep. (2016) 6:39541. 10.1038/srep39541 27996037 PMC5172230

[11] Corzo CA Condamine T Lu L Cotter MJ Youn JI Cheng P. HIF-1α regulates function and differentiation of myeloid-derived suppressor cells in the tumor microenvironment. J Exp Med. (2010) 207:2439–53. 10.1084/jem.20100587 20876310 PMC2964584

[12] Yang Q Li X Chen H Cao Y Xiao Q He Y. IRF7 regulates the development of granulocytic myeloid-derived suppressor cells through S100A9 transrepression in cancer. Oncogene. (2017) 36:2969–80. 10.1038/onc.2016.448 28092673

[13] Svoronos N Perales-Puchalt A Allegrezza MJ Rutkowski MR Payne KK Tesone AJ. Tumor cell-independent estrogen signaling drives disease progression through mobilization of myeloid-derived suppressor cells. Cancer Discov. (2017) 7:72–85. 10.1158/2159-8290.CD-16-0502 27694385 PMC5222699

[14] Ouyang LY Wu XJ Ye SB Zhang RX Li ZL Liao W. Tumor-induced myeloid-derived suppressor cells promote tumor progression through oxidative metabolism in human colorectal cancer. J Transl Med. (2015) 13:47. 10.1186/s12967-015-0410-7 25638150 PMC4357065

[15] Sun HL Zhou X Xue YF Wang K Shen YF Mao JJ. Increased frequency and clinical significance of myeloid derived suppressor cells in human colorectal carcinoma. World J Gastroenterol. (2012) 18:3303–9. 10.3748/wjg.v18.i25.3303 22783056 PMC3391769

[16] Srikrishna G. S100A8 and S100A9: new insights into their roles in malignancy. J Innate Immun. (2012) 4:31–40. 10.1159/000330095 21912088 PMC3250655

[17] Markowitz J Carson WE III. Review of S100A9 biology and its role in cancer. Biochim Biophys Acta. (2013) 1835:100–9. 10.1016/j.bbcan.2012.10.003 23123827 PMC3670606

[18] Laouedj M Tardif MR Gil L Raquil MA Lachhab A Pelletier M. S100A9 induces differentiation of acute myeloid leukemia cells through TLR4. Blood. (2017) 129:1980–90. 10.1182/blood-2016-09-738005 28137827

[19] Wu R Duan L Cui F Cao J Xiang Y Tang Y. S100A9 promotes human hepatocellular carcinoma cell growth and invasion through RAGE-mediated ERK1/2 and p38 MAPK pathways. Exp Cell Res. (2015) 334:228–38. 10.1016/j.yexcr.2015.04.008 25907296

[20] Ichikawa M Williams R Wang L Vogl T Srikrishna G. S100A8/A9 activate key genes and pathways in colon tumor progression. Mol Cancer Res. (2011) 9:133–48. 10.1158/1541-7786.MCR-10-0394 21228116 PMC3078037

[21] Duan L Wu R Ye LW Wang HY Yang X Zhang YY. S100A8 and S100A9 are associated with colorectal carcinoma progression and contribute to colorectal carcinoma cell survival and migration via Wnt/β-catenin pathway. PLoS ONE. (2013) 8:e62092. 10.1371/journal.pone.0062092 23637971 PMC3637369

[22] Chen B Miller AL Rebelatto M Brewah Y Rowe DC Clarke L. S100A9 induced inflammatory responses are mediated by distinct damage associated molecular patterns (DAMP) receptors *in vitro* and *in vivo*. PLoS ONE. (2015) 10:e0115828. 10.1371/journal.pone.0115828 25706559 PMC4338059

[23] Narumi K Miyakawa R Ueda R Hasimoto H Yamamoto Y Yoshida T. Proinflammatory proteins S100A8/S100A9 activate NK cells via interaction with RAGE. J Immunol. (2015) 194:5539–48. 10.4049/jimmunol.1402301 25911757

[24] Pruenster M Vogl T Roth J Sperandio M. S100A8/A9: From basic science to clinical application. Pharmacol Ther. (2016) 167:120–31. 10.1016/j.pharmthera.2016.07.015 27492899

[25] Wang SW Song R Wang ZY Jing Z Wang S Ma J. S100A8/A9 in Inflammation. Front Immunol. (2018) 9:1298. 10.3389/fimmu.2018.01298 29942307 PMC6004386

[26] Kim HJ Kang HJ Lee H Lee ST Yu MH Kim H. Identification of S100A8 and S100A9 as serological markers for colorectal cancer. J Proteome Res. (2009) 8:1368–79. 10.1021/pr8007573 19186948

[27] Shabani F Farasat A Mahdavi M Ghetbi N. Calprotectin (S100A8/S100A9): a key protein between inflammation and cancer. Inflamm Res. (2018) 67:801–12. 10.1007/s00011-018-1173-4 30083975

[28] Astrid R Julia N Peter A Jochen H The receptor RAGE: bridging inflammation and cancer. Cell Commun Signal. (2009) 7:12 10.1186/1478-811X-7-12 19426472 PMC2690588

[29] Xie JL Mendez JD Mendez-Valenzuela V Aguilar-Hernandez MM Cellular signaling of the receptor for advanced glycation end products (RAGE). Cell Signal. (2013) 25:2185–97. 10.1016/j.cellsig.2013.06.013 23838007

[30] Rahimifard M Maqbool F Moeini-Nodeh S Niaz K Abdollahi M Braidy N. Targeting the TLR4 signaling pathway by polyphenols: a novel therapeutic strategy for neuroinflammation. Ageing Res Rev. (2017) 36:11–9. 10.1016/j.arr.2017.02.004 28235660

[31] Blattner C Fleming V Weber R Himmelhan B Altevoqt P Gebhardt C. CCR5^+^ myeloid-derived suppressor cells are enriched and activated in melanoma lesions. Cancer Res. (2017) 78:157–67. 10.1158/0008-5472.CAN-17-0348 29089297

[32] Yu SJ Ma C Heinrich B Brown ZJ Sandhu M Zhang Q. Targeting the crosstalk between cytokine-induced killer cells and myeloid-derived suppressor cells in hepatocellular carcinoma. J Hepatol. (2019) 70:449–57. 10.1016/j.jhep.2018.10.040 30414862 PMC6380944

[33] Waiqht JD Netherby C Hensen ML Miller A Hu Q Liu S Myeloid-derived suppressor cell development is regulated by a STAT/IRF-8 axis. J Clin Invest. (2013) 123:4464–78. 10.1172/JCI68189 24091328 PMC3784535

[34] Mace TA Ameen Z Collins A Wojcik S Mari M Young GS. Pancreatic cancer-associated stellate cells promote differentiation of myeloid-derived suppressor cells in a STAT3-dependent manner. Cancer Res. (2013) 73:3007–18. 10.1158/0008-5472.CAN-12-4601 23514705 PMC3785672

[35] Lechner MG Liebertz DJ Epstein AL. Characterization of cytokine induced myeloid-derived suppressor cells from normal human peripheral blood mononuclear cells. J Immunol. (2010) 185:2273–84. 10.4049/jimmunol.1000901 20644162 PMC2923483

[36] Ostrand-Rosenberg S Sinha P. Myeloid-derived suppressor cells: linking inflammation and cancer. J Immunol. (2009) 182:4499–506. 10.4049/jimmunol.0802740. 19342621 PMC2810498

[37] Sinha P Okoro C Foell D Freese HH Ostrand-Rosenberg S Srikrishna G. Proinflammatory S100 proteins regulate the accumulation of myeloid-derived suppressor cells. J Immunol. (2008) 181:4666–75. 10.4049/jimmunol.181.7.4666 18802069 PMC2810501

[38] Zhao F Hoechst B Duffy A Gamrekelashvili J Fioravanti S Manns MP. S100A9 a new marker for monocytic human myeloid-derived suppressor cells. Immunology. (2012) 136:176–83. 10.1111/j.1365-2567.2012.03566.x 22304731 PMC3403264

[39] Bayne LJ Beatty GL Jhala N Clark CE Rhim AD Stanger BZ. Tumor-derived granulocyte-macrophage colony-stimulating factor regulates myeloid inflammation and T cell immunity in pancreatic cancer. Cancer Cell. (2012) 21:822–35. 10.1016/j.ccr.2012.04.025 22698406 PMC3575028

[40] Chang AL Miska J Wainwright DA Dey M Rivetta CV Yu D. CCL2 produced by the glioma microenvironment is essential for the recruitment of regulatory T cells and myeloid-derived suppressor cells. Cancer Res. (2016) 76:5671–82. 10.1158/0008-5472.CAN-16-0144 27530322 PMC5050119

[41] Obermajer N Muthuswamy R Odunsi K Edwards RP Kalinski P PGE2-induced CXCL12 production and CXCR4 expression controls the accumulation of human mdscs in ovarian cancer environment. Cancer Res. (2011) 71:7463–70. 10.1158/0008-5472.CAN-11-2449 22025564 PMC4993027

[42] Alfaro C Teijeira A Onate C Perez G Sanmamed MF Andueza MP. Tumor-produced interleukin-8 attracts human myeloid-derived suppressor cells and elicits extrusion of neutrophil extracellular traps (NETs). Clin Cancer Res. (2016) 22:3924–36. 10.1158/1078-0432.CCR-15-2463 26957562

[43] Ryckmam C Vandal K Rouleau P Talbot M Tessier PA Proinflammatory activities of S100: proteins S100A8, S100A9, and S100A8/A9 induce neutrophil chemotaxis and adhesion. J Immunol. (2003) 170:3233–42. 10.4049/jimmunol.170.6.3233 12626582

[44] Lee TH Chang HS Bae DJ Song HJ Kim MS Park JS. Role of S100A9 in the development of neutrophilic inflammation in asthmatics and in a murine model. Clin Immunol. (2017) 183:158–66. 10.1016/j.clim.2017.08.013 28847516

[45] Tsai SY Segovia JA Chang TH Morris LR Berton MT Tessier PA. DAMP molecule S100A9 acts as a molecular pattern to enhance inflammation during influenza A virus infection: role of DDX21-TRIF-TLR4-MyD88 pathway. PLoS Pathog. (2014) 10:e1003848. 10.1371/journal.ppat.1003848 24391503 PMC3879357

[46] Vogl T Stratis A Wixler V Voller T Thurainayagam S Jorch SK. Autoinhibitory regulation of S100A8/S100A9 alarmin activity locally restricts sterile inflammation. J Clin Invest. (2018) 128:1852–66. 10.1172/JCI89867 29611822 PMC5919817

[47] Okada K Arai S Itoh H Adachi S Hayashida M Nakase H. CD68 on rat macrophages binds tightly to S100A8 and S100A9 and helps to regulate the cells immune functions. J Leukoc Biol. (2016) 100:1093–104. 10.1189/jlb.2A0415-170RRR 27312849

