# Supplementary material for: S100A9 Regulates MDSCs-Mediated Immune Suppression via the RAGE and TLR4 Signaling Pathways in Colorectal Carcinoma
Source: Front Immunol. 2019 Sep 18;10:2243. doi: 10.3389/fimmu.2019.02243 (PMC6759487; doi:10.3389/fimmu.2019.02243)
Supplement: Supplementary file 1 [file Image_1.pdf]

Supplementary material

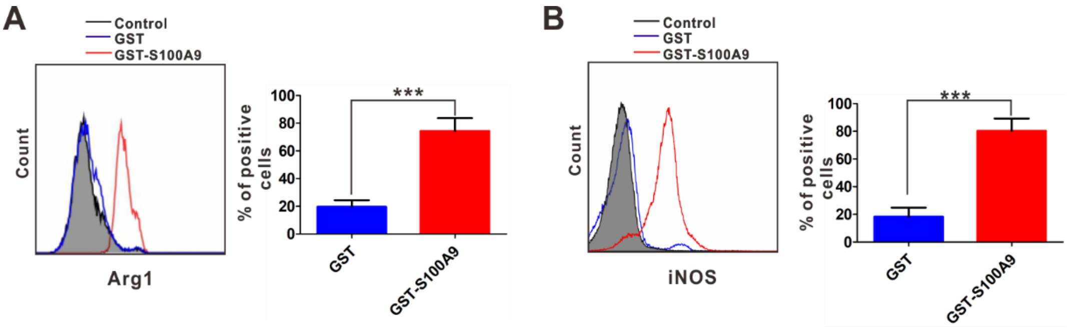

FIGURE S1 | Representative histograms for intracellular immunosuppressive molecules Arg1(A) and iNOS (B) expression in MDSCs by FCM analysis. The gray-filled histograms represent controls, and the different colored dotted lines represent MDSCs treated with GST-S100A9 (20μg/ml) and GST (20μg/ml) proteins. Statistical analysis of the mean percentages of Arg-1 (A) and iNOS (B) in positive cells in MDSC population. Data represents the mean±SD. ***$p<0.001$.
